# Supplementary material for: The chimeric TAC receptor co-opts the T cell receptor yielding robust anti-tumor activity without toxicity
Source: Nat Commun. 2018 Aug 3;9:3049. doi: 10.1038/s41467-018-05395-y (PMC6076291; doi:10.1038/s41467-018-05395-y)
Supplement: Supplementary file 1 — Supplementary Information [file 41467_2018_5395_MOESM1_ESM.pdf]

The chimeric TAC receptor co-opts the T cell receptor yielding robust anti-tumor activity without toxicity

Helsen et al.

Supplementary Figures

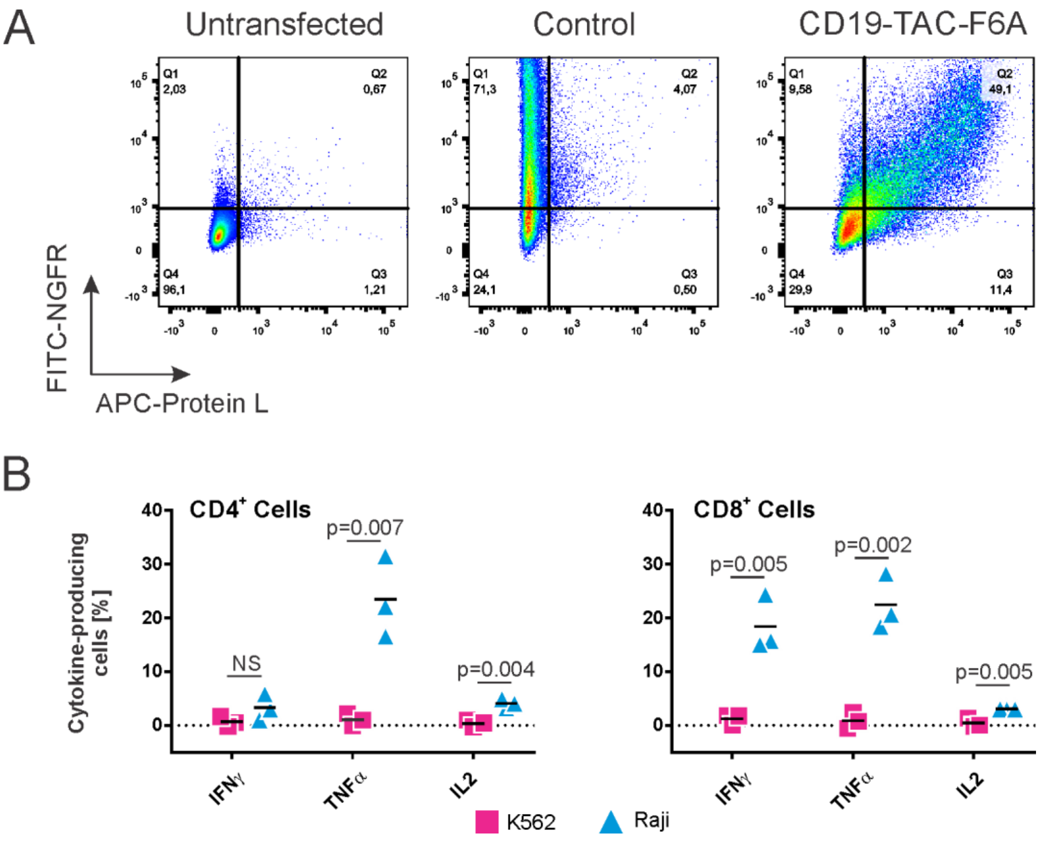

Supplementary Figure 1:

F6A scFv TAC can be detected by Protein L and F6A TAC engineered T cells show antigen specific reactivity. **A.** Protein L binds the kappa light chain of scFv. HEK 293T cells were transfected with CD19-TAC-F6A, stained and analyzed for TAC and tNGFR expression by flow cytometry. For the gating strategy see Supplementary Fig. 16. **B.** CD19-TAC-cells were stimulated with antigen-positive Raji (triangle) or antigen-negative K562 (square) tumor cells, respectively. Data are presented as percent of CD4 or CD8 T cells producing cytokine. Lines represent data medians. Multiple t-test was used to determine significance. For the gating strategy see Supplementary Fig. 13B.

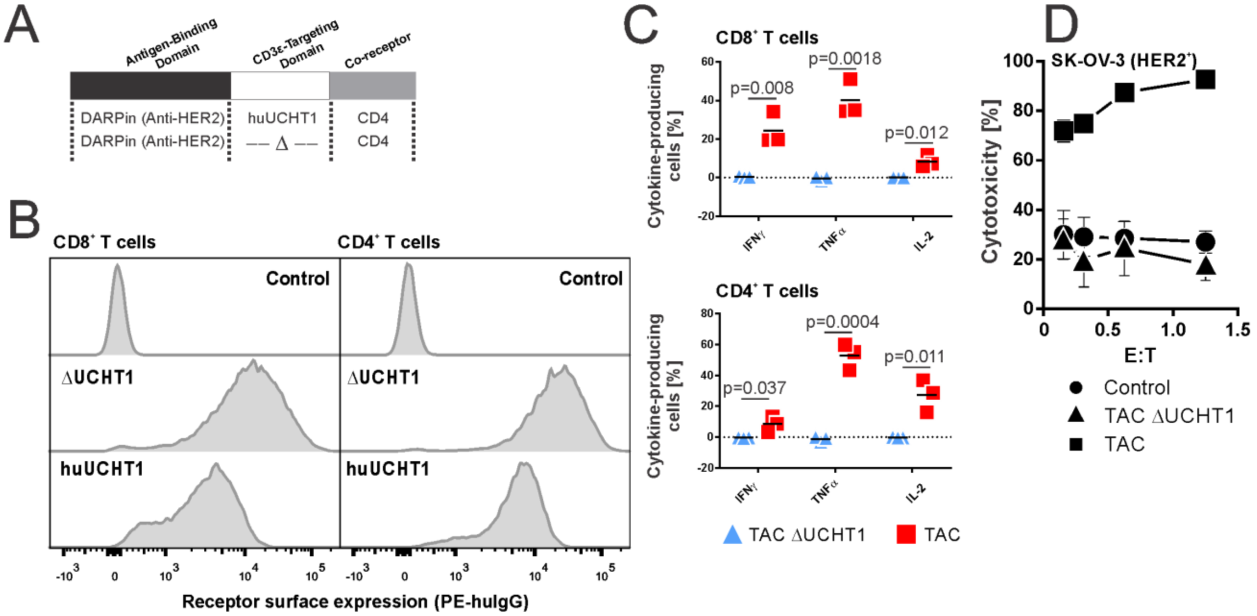

Supplementary Figure 2:

CD3-binding domain is required for TAC-engineered T cell function. Full-length and ΔUCHT1 TAC receptors (**A**) were expressed on the surface of primary human T cells (**B**). Relative TAC surface expression is measured by flow cytometry. Cells were stained for CD4, CD8, tNGFR and TAC (via its Myc Tag), and gated on either $CD4^+NGFR^+$ or $CD8^+NGFR^+$; representative TAC expression data are presented as histograms. For the gating strategy see Supplementary Fig. 13A. **C**. HER2-TAC-T cells (bearing huUCHT1 (square) or ΔUCHT1 (triangle)) are stimulated with antigen-positive SK-OV-3 tumor cells. Data are presented as percent of $CD4^+$ or $CD8^+$ T cells producing cytokine. Lines represent data means. Multiple t-test was used to determine significance. For the gating strategy see Supplementary Fig. 13B. **D**. HER2-TAC-T cells (bearing huUCHT1 (square) or ΔUCHT1 (triangle)) and vector control T cells (circles) are co-cultured with SK-OV-3 tumor cells to measure TAC-T cell-mediated cytotoxicity. Data are from 3 independent experiments with 3 different donors; error bars show standard deviation.

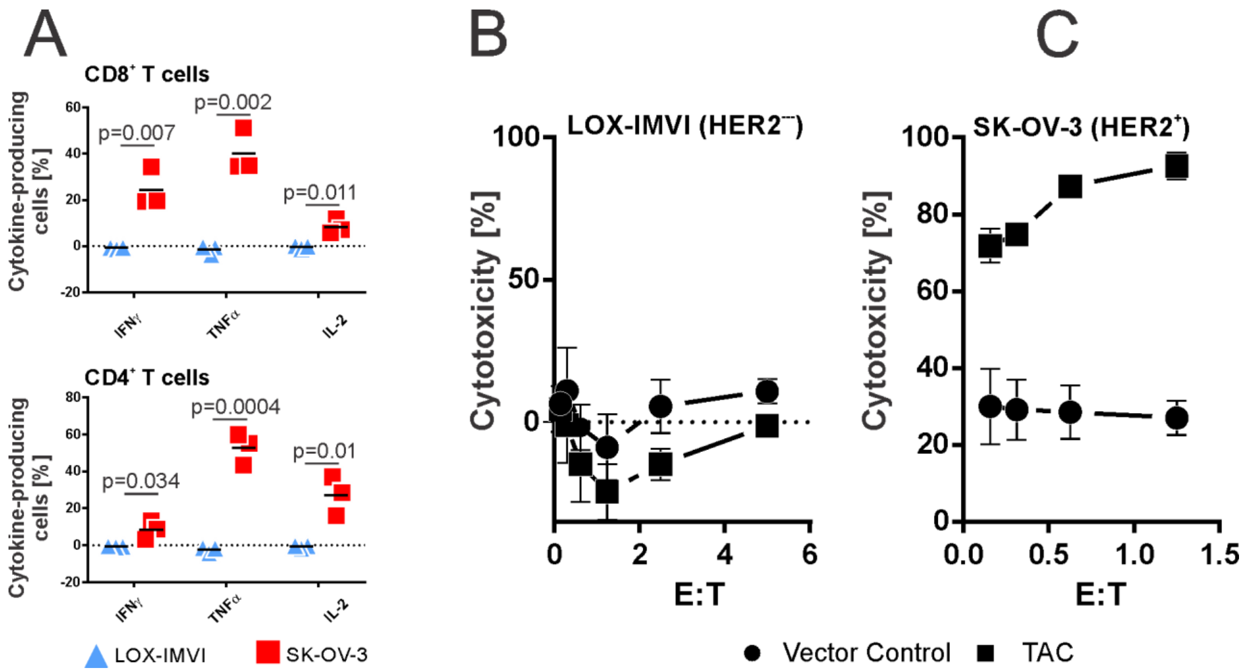

Supplementary Figure 3:

TAC-T cells show no evidence of auto-activation in the absence of target antigen. Data originates from the same experiment as Supplemental Figure 2. **A**. HER2-TAC-T cells are stimulated with antigen-positive SK-OV-3 (square) or antigen-negative LOX-IMVI (triangle) tumor cells. Data are presented as percent of $CD4^+$ or $CD8^+$ T cells producing cytokine. Lines represent data means. Multiple t-test was used to determine significance. For the gating strategy see Supplementary Fig. 13B. **B-C**. HER2-TAC-T cells (bearing UCHT1; square) and vector control T cells (circle) are co-cultured with LOX-IMVI or SK-OV-3 tumor cells to measure TAC-T cell-mediated cytotoxicity. Data are from 3 independent experiments with 3 different donors; error bars show standard deviation.

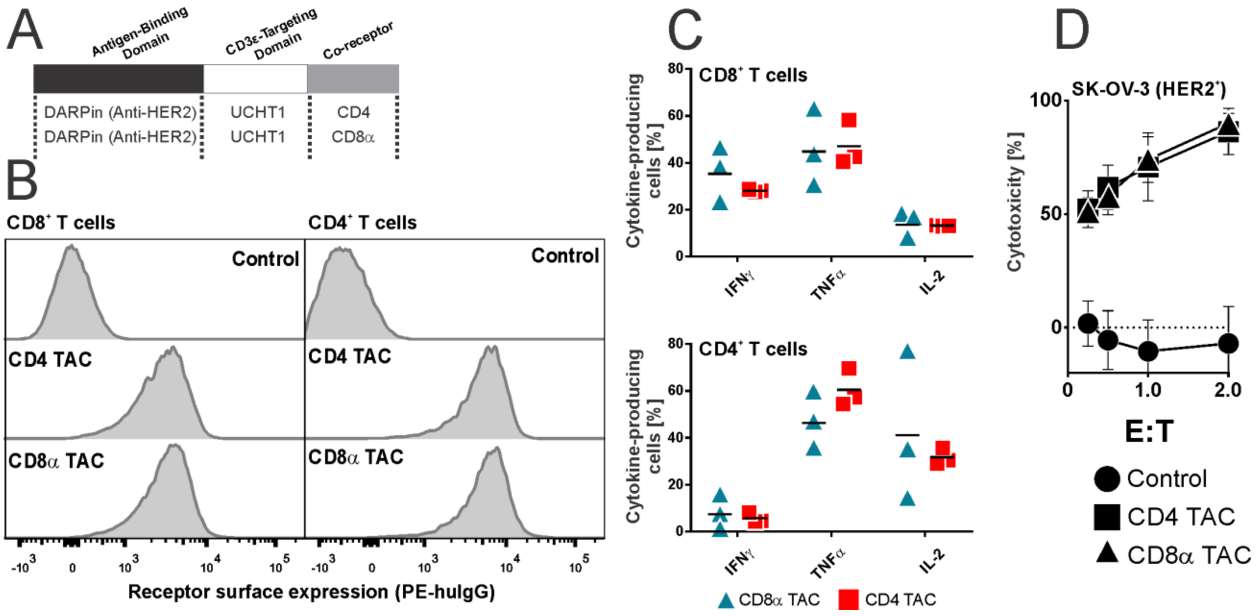

Supplementary Figure 4:

Evaluation of cytosolic TAC domains. **A**. Schematic representation of CD4 and CD8α TAC constructs containing the anti-HER2 DARPin and UCHT1 CD3-binding domain. **B**. Cells were stained for CD4, CD8, tNGFR, and TAC expression, and gated on either $CD4^+NGFR^+$ or $CD8^+NGFR^+$; representative TAC expression data are presented as histograms. For the gating strategy see Supplementary Fig. 13A. **C**. Cytokine production by CD4 TAC- (square) and CD8α TAC- (triangle) T cells stimulated by $HER2^+$ SK-OV-3 tumor cells are shown. Lines represent data means. Multiple t-test was used to determine significance. For the gating strategy see Supplementary Fig. 13B. **D.** Cytotoxicity was measured by co-culturing $HER2^+$ SK-OV-3 tumor cells with TAC- (CD4 co-receptor (squares) or CD8α co-receptor (triangles)) or vector control (circles) T cells. Data are from 3 independent experiments with 3 different donors; error bars show standard deviation.

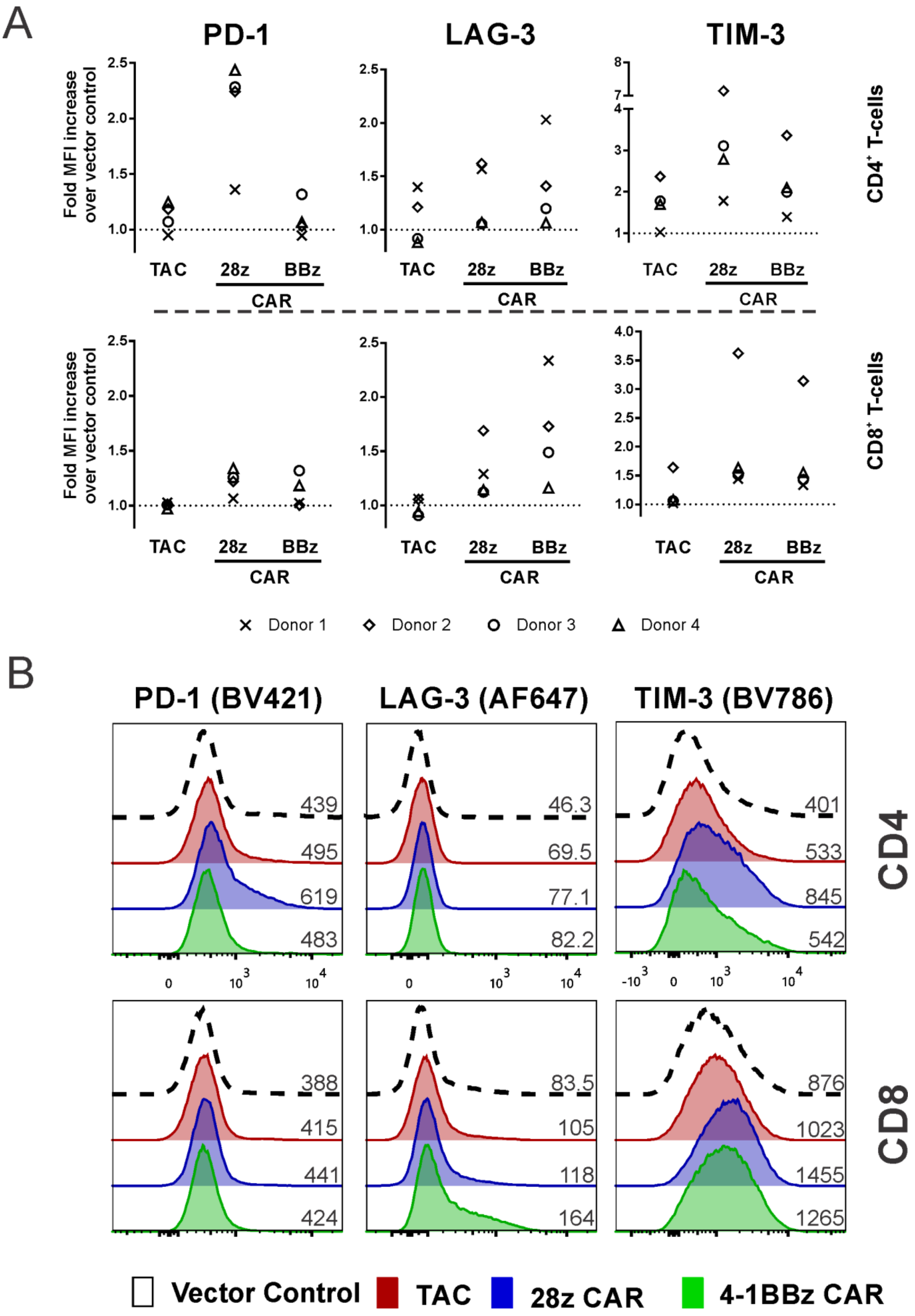
A
PD-1
LAG-3
TIM-3
Fold MFI increase over vector control
TAC
28z
BBz
CAR
CD4+ T-cells
CD8+ T-cells
Donor 1
Donor 2
Donor 3
Donor 4
B
PD-1 (BV421)
LAG-3 (AF647)
TIM-3 (BV786)
439
495
619
483
46.3
69.5
77.1
82.2
401
533
845
542
CD4
388
415
441
424
83.5
105
118
164
876
1023
1455
1265
CD8
Vector Control
TAC
28z CAR
4-1BBz CAR

Supplementary Figure 5:

Relative expression of checkpoint receptors in CAR- and TAC-engineered T cells. T cells are transduced with HER2-TAC, anti-HER2 28ζ CAR or anti-HER2 BBζ CAR, stained for surface marker expression, and analyzed by flow cytometry. **A.** Expression of checkpoint receptors PD-1, LAG-3 and TIM-3. Populations are gated on either $CD4^+NGFR^+$ or $CD8^+NGFR^+$. All data are normalized to vector control (vector only carrying tNGFR). Data is derived from 4 individual donors across two separate experiments. Each donor is represented by a unique symbol to visualize donor-to-donor variations. **B.** Representative histograms of HER2-TAC (red), anti-HER2 28ζ CAR (blue) or anti-HER2 BBζ CAR (green). Populations are gated on either $CD4^+NGFR^+$ or $CD8^+NGFR^+$. Representative, normalized histograms per marker are shown. Values shown indicate mean fluorescence intensity. For both A and B the gating strategy is shown in Supplementary Fig. 14.

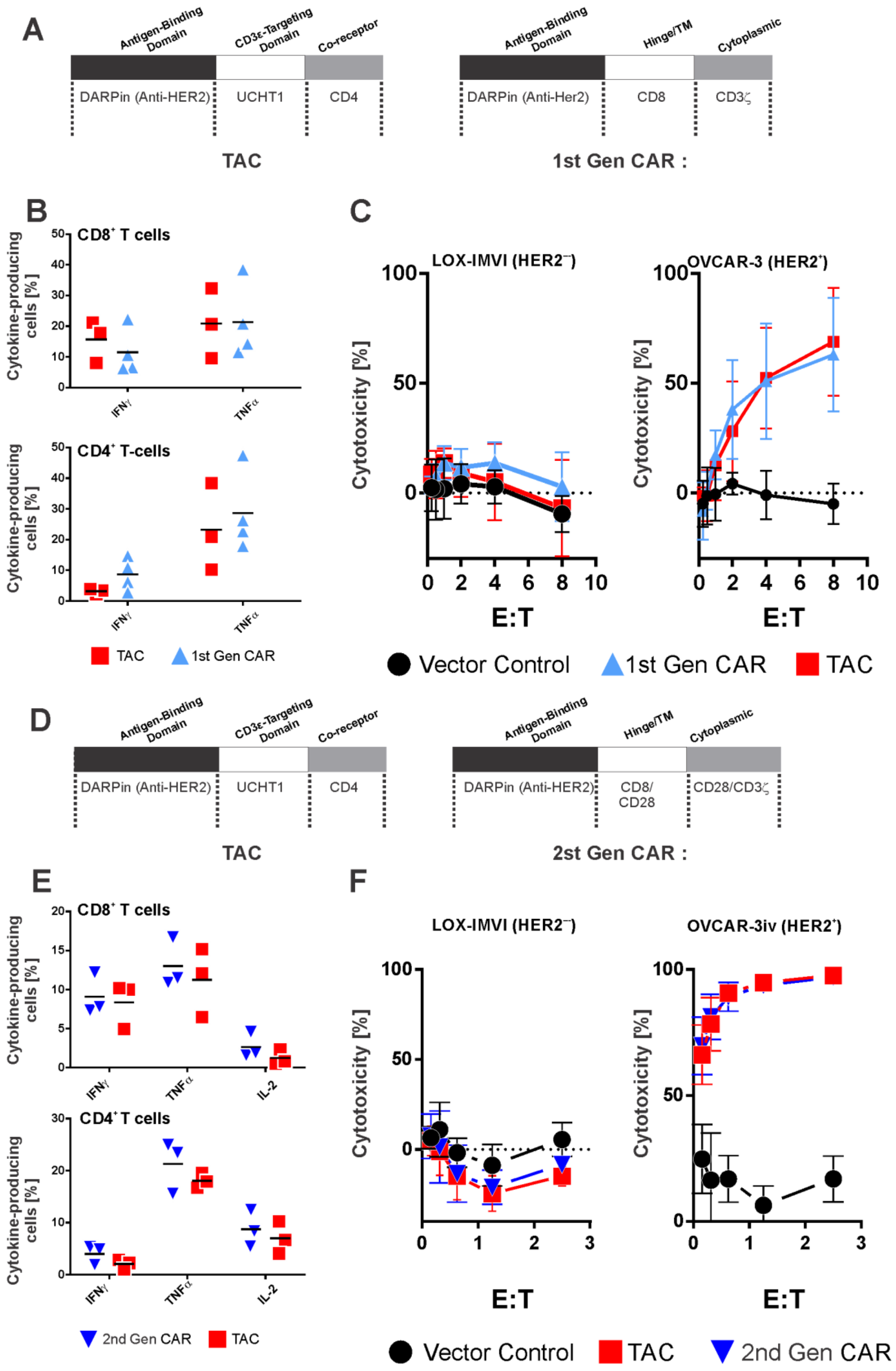
A
Antigen-Binding Domain
CD3ε-Targeting Domain
Co-receptor
DARPin (Anti-HER2)
UCHT1
CD4
TAC
Antigen-Binding Domain
Hinge/TM
Cytoplasmic
DARPin (Anti-Her2)
CD8
CD3ζ
1st Gen CAR :
B
CD8+ T cells
Cytokine-producing cells [%]
IFNγ
TNFα
CD4+ T-cells
TAC
1st Gen CAR
C
LOX-IMVI (HER2⁻)
OVCAR-3 (HER2+)
Cytotoxicity [%]
E:T
Vector Control
1st Gen CAR
TAC
D
Antigen-Binding Domain
CD3ε-Targeting Domain
Co-receptor
DARPin (Anti-HER2)
UCHT1
CD4
TAC
Antigen-Binding Domain
Hinge/TM
Cytoplasmic
DARPin (Anti-HER2)
CD8/ CD28
CD28/CD3ζ
2st Gen CAR :
E
CD8+ T cells
Cytokine-producing cells [%]
IFNγ
TNFα
IL-2
CD4+ T cells
2nd Gen CAR
TAC
F
LOX-IMVI (HER2⁻)
OVCAR-3iv (HER2+)
Cytotoxicity [%]
E:T
Vector Control
TAC
2nd Gen CAR

Supplementary Figure 6:

First-generation CAR-, second-generation CAR-, and TAC-T cells exhibit similar *in vitro* potency. A. Schematics of TAC and first-generation CAR constructs. **B.** Comparison of cytokine production from $CD4^+$ or $CD8^+$ TAC- (square) and first-generation CAR- (triangle) T cells when stimulated with $HER2^+$ OVCAR-3 tumor cells (minus cytokine production triggered by $HER2^–$ LOX-IMVI tumor cells). Lines represent data means. **C.** Cytotoxicity of TAC- (square) and first-generation CAR- (triangle) relative to vector control (circle) T cells against OVCAR-3 and LOX-IMVI tumor cells. Data are from 3-4 independent experiments; error bars show standard error of the mean. **D.** Schematics of the TAC and second-generation CD28-based CAR constructs. **E.** Comparison of cytokine production from $CD4^+$ or $CD8^+$ TAC- (square) and second-generation CAR- (inverted triangle) T cells when stimulated with OVCAR-3 (minus cytokine production triggered by $HER2^–$ LOX-IMVI tumor cells). Lines represent data means. **F.** Cytotoxicity of TAC- (square) and second-generation CD28-based CAR- (inverted triangle) relative to vector control (circle) T cells against LOX-IMVI and OVCAR-3 tumor cells. Data are from 3 independent experiments; error bars show standard error of the mean. For B and E the gating strategy is shown if Supplementary Fig. 13B.

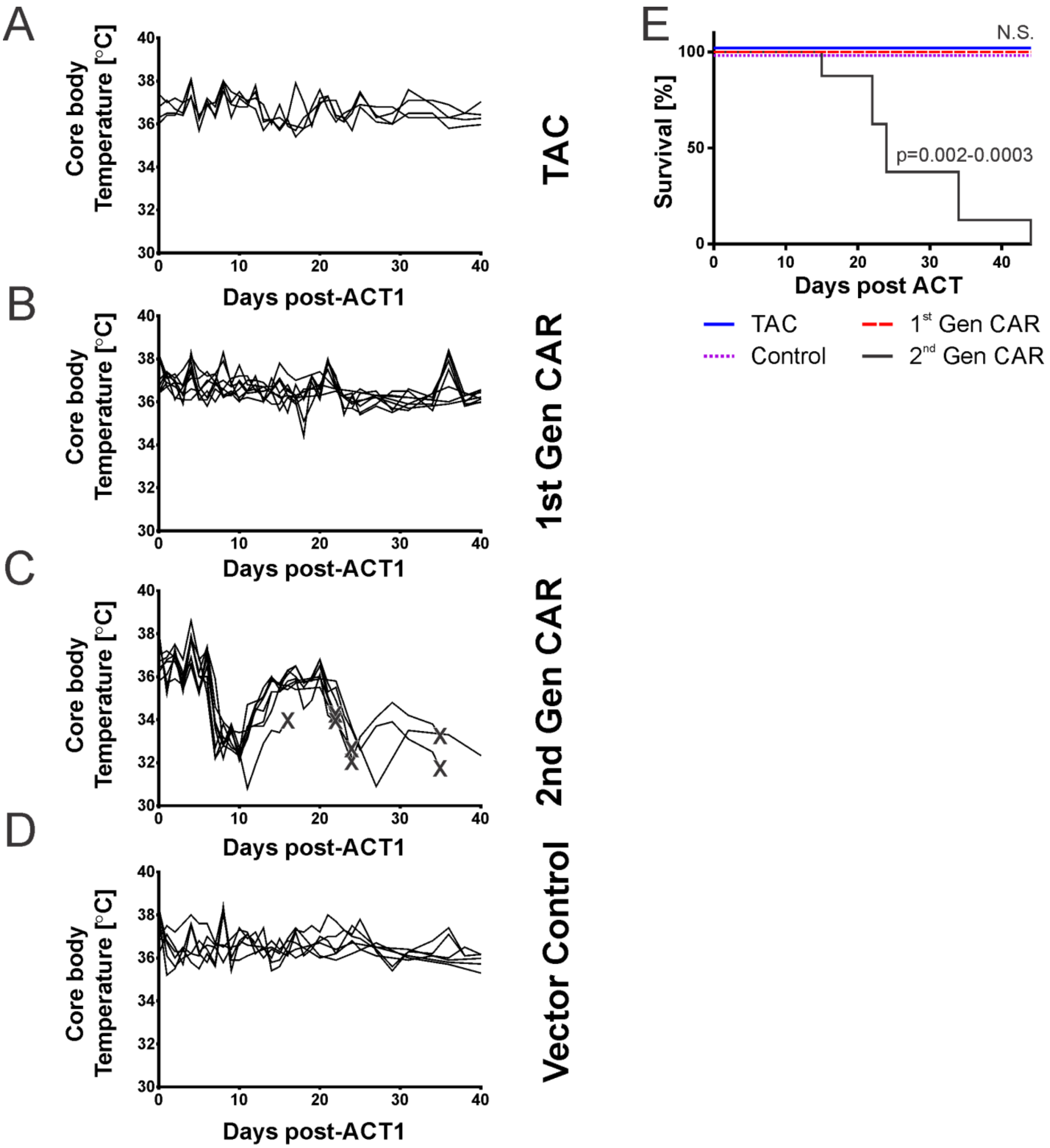

A
B
C
D
E
Core body Temperature [°C]
Days post-ACT1
TAC
1st Gen CAR
2nd Gen CAR
Vector Control
Survival [%]
Days post ACT
N.S.
p=0.002-0.0003
TAC
1st Gen CAR
Control
2nd Gen CAR

Supplementary Figure 7:

HER2-TAC-T cells demonstrate an enhanced safety profile over first- and second-generation HER2-CAR-T cells *in vivo*. OVCAR-3 tumor-bearing mice are treated with 2.0×10^6 HER2-TAC-T cells (**A**), first-generation anti-HER2 CAR-T cells (**B**), second-generation anti-HER2 28ζ CAR-T cells (**C**), or a matched total number of vector control T cells (**D**). Mice are followed for change in core body temperature; each curve displays data from a single treated mouse. When mice reach a toxicity-induced endpoint, this is indicated via an X. Data has been replicated in an additional independent experiment. The change in core body temperature between vector control, 1st gen CAR, and TAC was not significantly different. Significance was determined via curve fitting and multiple t-test. Survival is shown in (**E**). Vector control (purple, dotted), TAC (blue, solid) or first-generation CAR (red, dashes) treated mice showed no toxicity-induced endpoints and were not significantly different. In contrast, second-generation 28ζ CAR (black, solid)-treated mice all reached toxicity-induced endpoint within 44 days. Mice were not followed to tumor volume endpoint. Significance was determined via the Log Rank test.

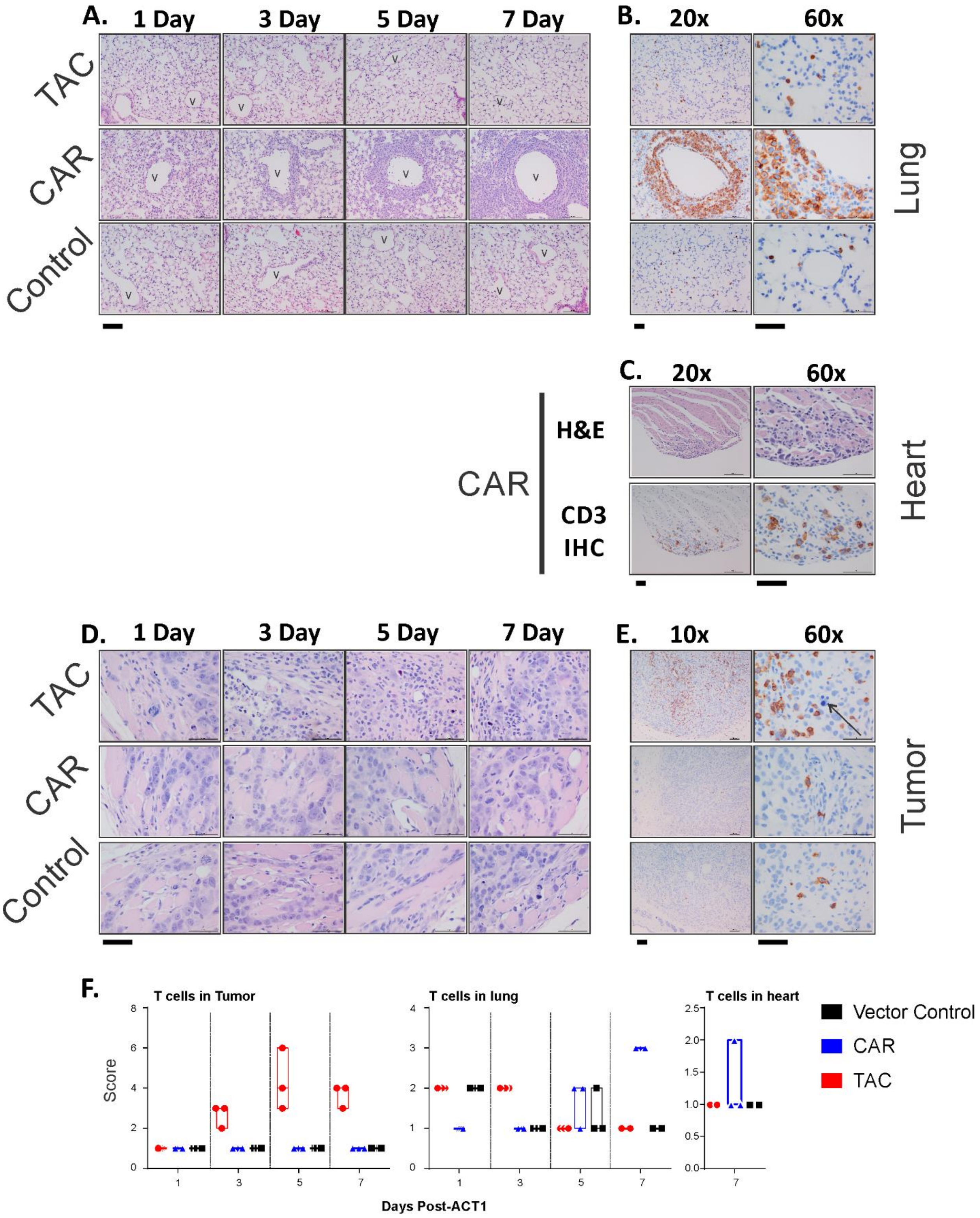
A.
1 Day
3 Day
5 Day
7 Day
TAC
CAR
Control
B.
20x
60x
Lung
C.
20x
60x
CAR
H&E
CD3
IHC
Heart
D.
1 Day
3 Day
5 Day
7 Day
TAC
CAR
Control
E.
10x
60x
Tumor
F.
T cells in Tumor
T cells in lung
T cells in heart
Score
Days Post-ACT1
Vector Control
CAR
TAC

Supplementary Figure 8:

TAC- and CAR-T cells show differential patterns of localization *in vivo*. OVCAR-3 tumor-bearing mice were treated with 6.0×10^6 second-generation anti-HER2 28ζ CAR- or HER2-TAC-T cells, or a matched total number of vector control T cells. At 1, 3, 5, and 7 days post-ACT1 mice (n = 3 per treatment) were perfused and tissues were formalin fixed and paraffin embedded. **A.** Timecourse of H&E (hematoxylin and eosin) stained lung sections at 20X magnification; vasculature is indicated by a "v". Scale bar indicates 100 μm. **B.** CD3 IHC staining of lung sections at 7 days post-ACT1. Scale bar indicates 50 μm**. C.** H&E and CD3 IHC of cardiac tissue at 7 days post-ACT1. Scale bar indicates 50 μm. **D.** Timecourse of H&E stained tumor sections at 60X magnification. Scale bar indicates 50 μm. **E.** CD3 IHC staining of tumor sections at 7 days post-ACT1; arrow indicates a necrotic tumor cell. Scale bar indicates 50 μm. In all cases, images are representative of observations in all mice (n = 3 each). **F.** CD3 IHC was performed on heart, lung, and tumor tissue; T cell infiltrate was scored as % infiltrate based on tissue area in 10% intervals (score 1 = <1%, score 2 = 1-10%, score 3 = 10-20%, score 4 = 20-30%, score 5 = 30-40%, and score 6 = 40-50%). Data presented as an average score for n = 3 mice per time point.

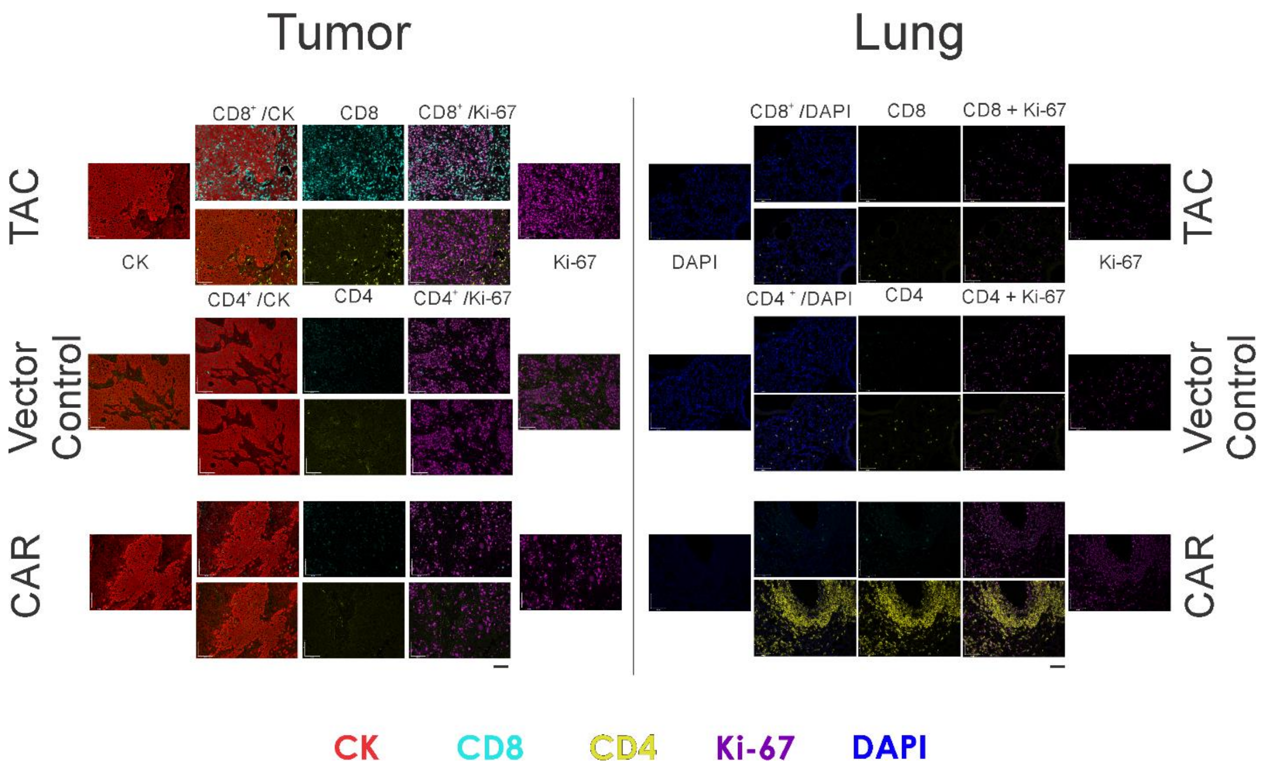

Supplementary Figure 9:

Single color and composite multicolor IHC tissue analysis. OVCAR-3 tumor-bearing mice were treated with 6.0×10^6 second-generation anti-HER2 28ζ CAR- or HER2-TAC-T cells, or a matched total number of vector control T cells. At 7 days post-ACT1 mice (n = 3 per treatment) were perfused and tissues were formalin fixed and paraffin embedded for subsequent multicolor IHC analysis (tumor or lung tissues were stained for human cytokeratin (CK, red), cellular proliferation marker Ki-67 (purple), CD8 (cyan), CD4 (yellow), and DAPI (blue)). Representative single-color images are shown alongside 2-color overlay images. Scale bar indicates 100 μm.

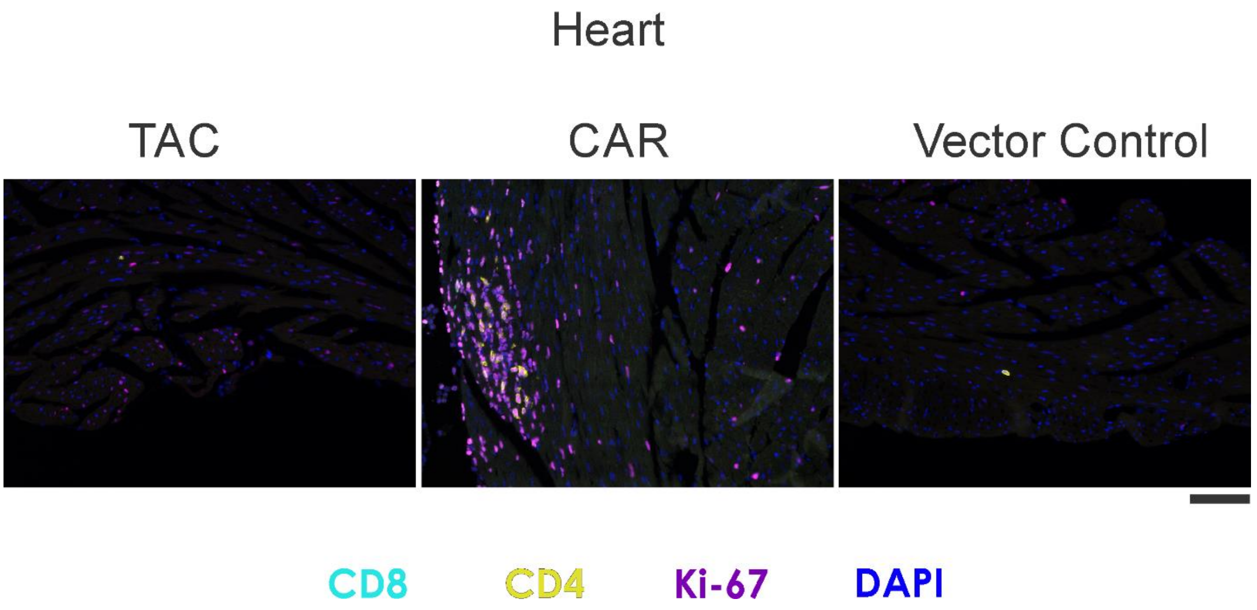

Supplementary Figure 10:
Multicolor IHC of cardiac tissue at 7 days post-ACT1. OVCAR-3 tumor bearing mice were treated with 6.0×10^6 second-generation anti-HER2 28ζ CAR- or HER2-TAC-T cells, or a matched total number of vector control T cells. At 7 days post-ACT1 mice (n = 3 per treatment) were perfused and tissues were formalin fixed and paraffin embedded for subsequent multicolor IHC analysis (cardiac tissue was stained for cellular proliferation marker Ki-67 (purple), CD8 (cyan), CD4 (yellow), and DAPI (blue)). Scale bar indicates 100 μm. Representative images are shown.

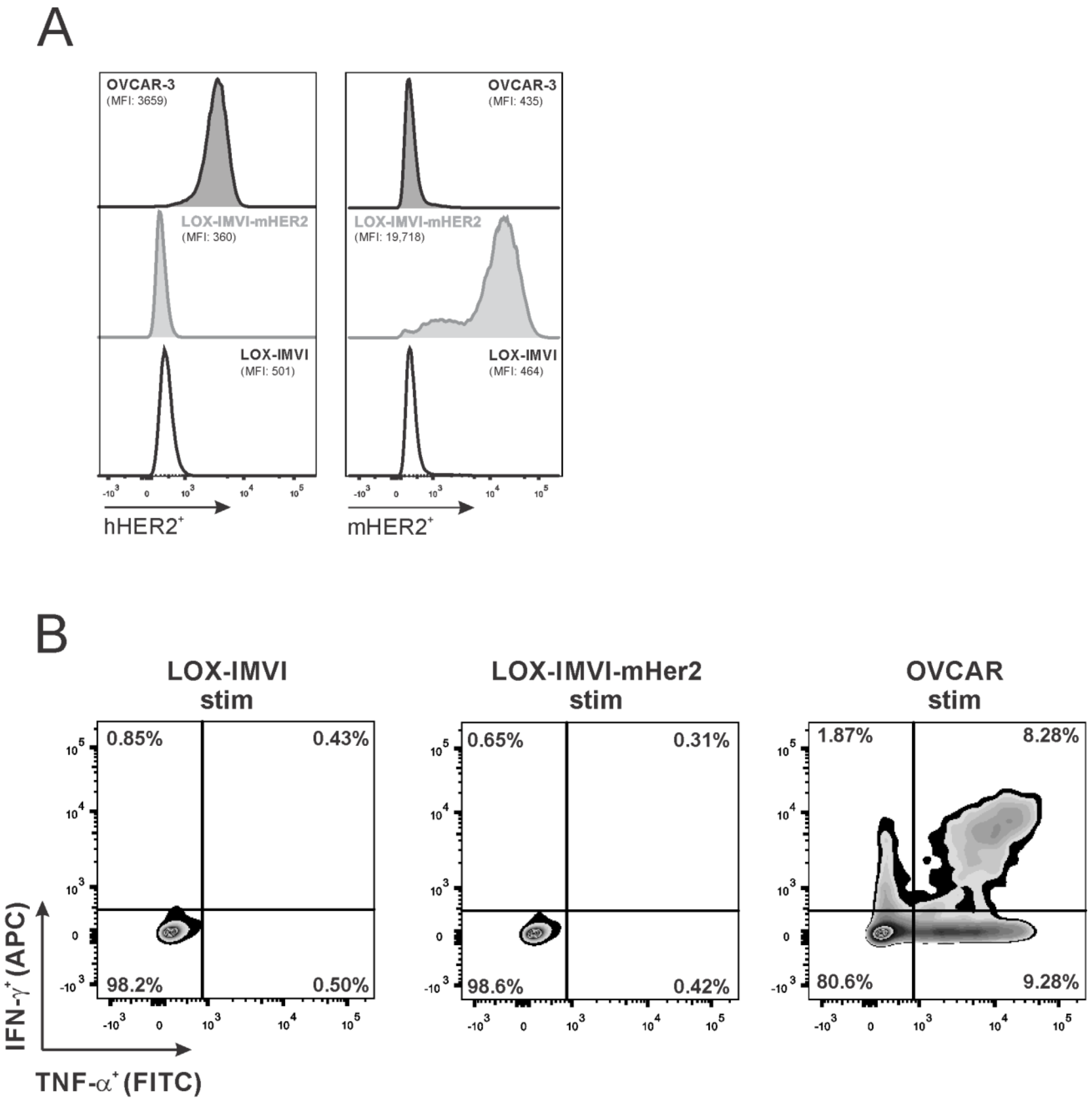

Supplementary Figure 11**.**

Second generation anti-HER2 28ζ CAR-T cells do not respond to murine HER2. A. Expression of human or murine HER2 (hHER2 or mHER2, respectively) on OVCAR-3, LOX-IMVI, and LOX-IMVI-mHER2 tumor cell lines as determined by flow cytometry. For the gating strategy see Supplementary Fig. 16. **B.** Production of activation cytokines (IFN-γ and TNF-α) by $CD8^+$ CD28ζ-CAR T cells after stimulation with tumor cell lines (as indicated) was determined by flow cytometry. Data is representative of findings from two independent experiments. For the gating strategy see Supplementary Fig. 13B.

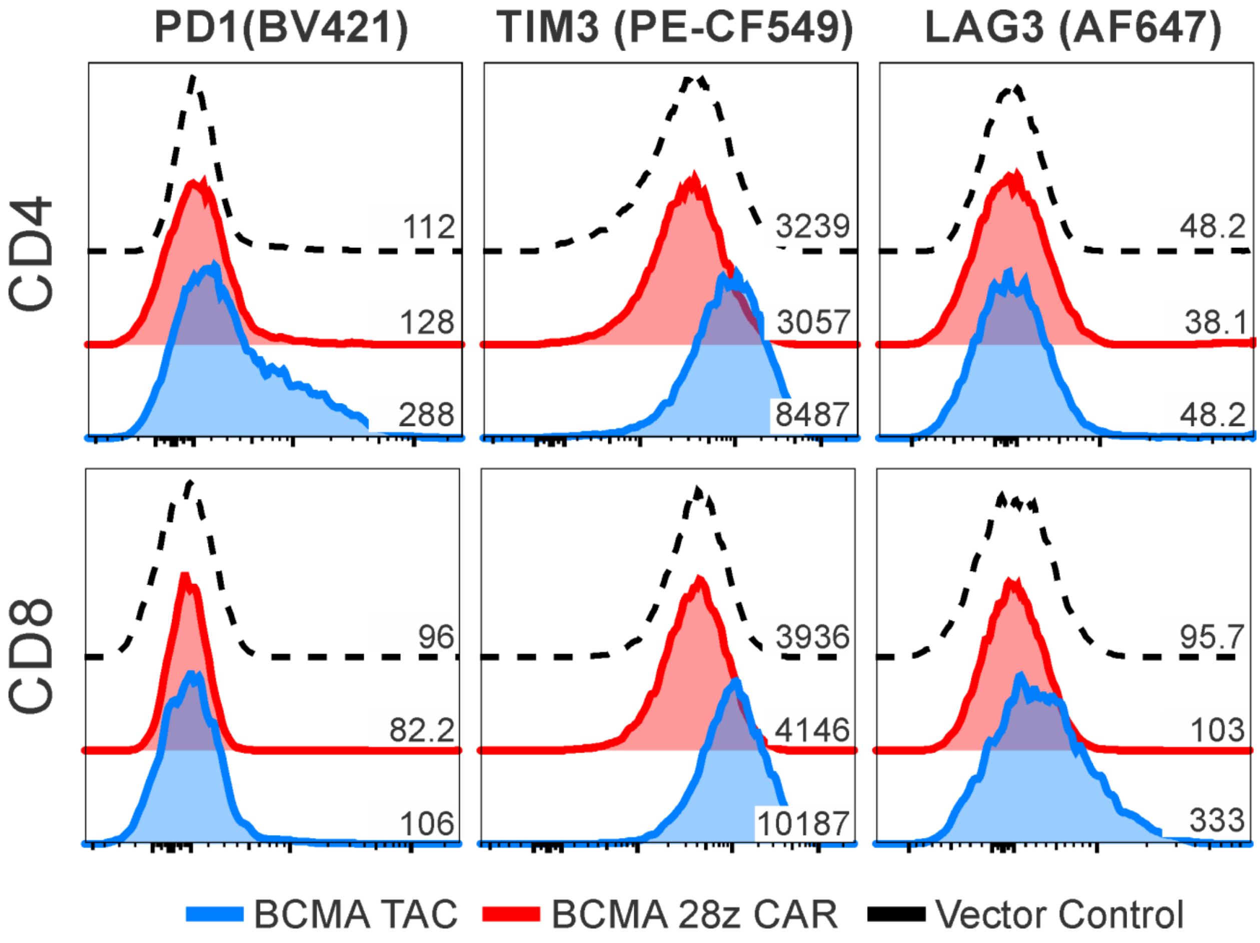

Supplementary Figure 12:
Relative expression of checkpoint receptors in BCMA-CAR- and TAC-engineered T cells.
T cells were transduced with BCMA-TAC, second-generation BCMA-CD28ζ-CAR, or vector control (tNGFR). Engineered T cells are stained for surface marker expression and analyzed by flow cytometry. Populations are gated on $CD4^{+}NGFR^{+}$ or $CD8^{+}NGFR^{+}$ analysis. Values indicate MFI of each marker. Histograms are from one donor, representative of three donors from three independent experiments. For the gating strategy see Supplementary Fig. 14

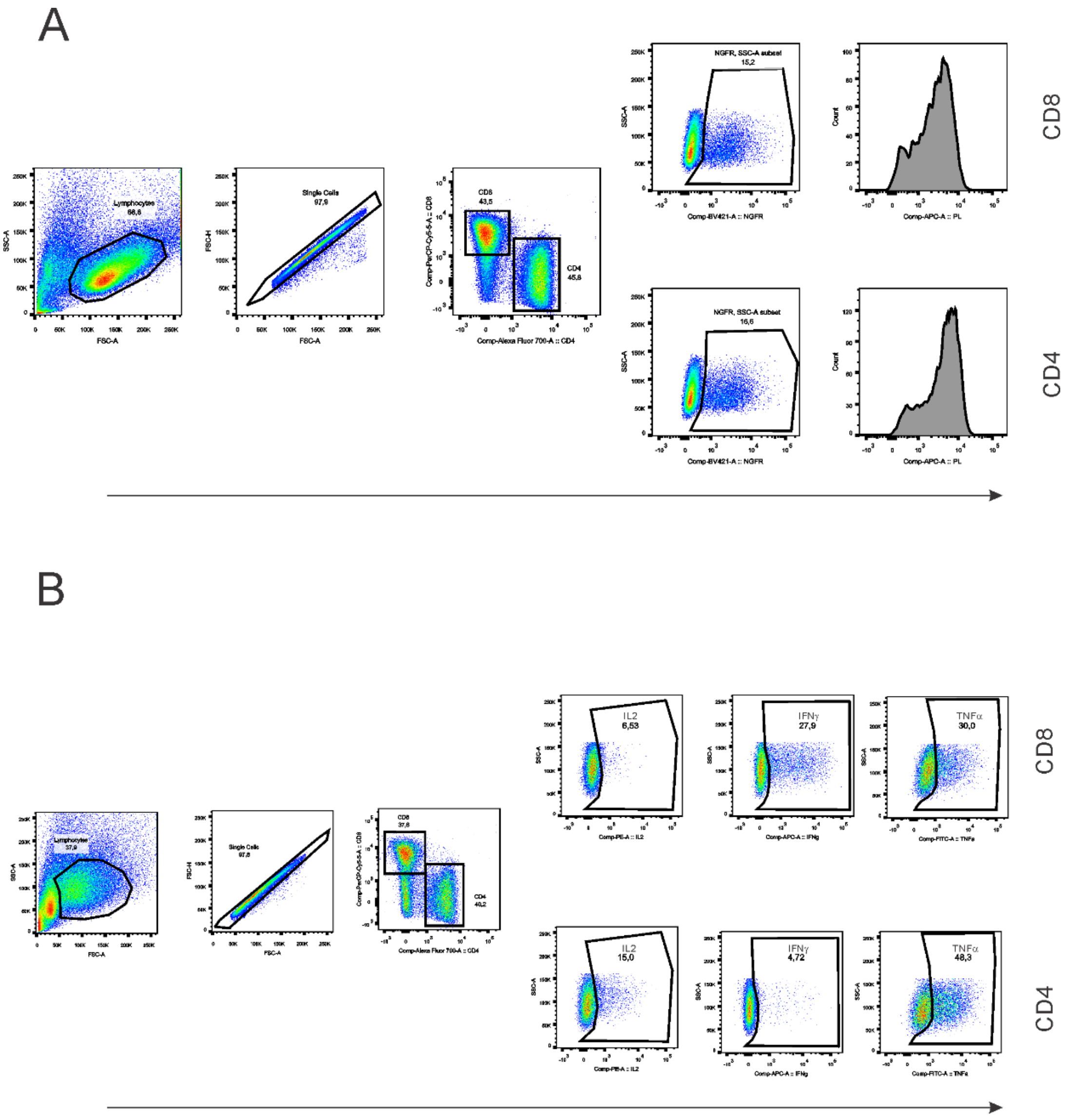

Supplementary Figure 13:

Examples of gating strategies used for the analysis of flow cytometry data. A. Gating strategy for phenotypic analysis of engineered T cells. Lymphocytes (SSC-A v. FSC-A) → singlets (FSC-H v. FSC-A) → $CD8^+$ *or* $CD4^+$ cells (CD8-PerCPCy5.5 v. CD4-AF700) → $NGFR^+$ cells (SSC-A v. NGFR-BV421) → TAC receptor expression as a histogram (Protein L^+ with indirect detection via APC-conjugated streptavidin is shown as an example). $NGFR^+$ gates were set based on fully stained, non-transduced T cell controls. **B.** Gating strategy for functional cytokine production by tumor cell line-stimulated engineered T cells. Lymphocytes (SSC-A v. FSC-A) → singlets (FSC-H v. FSC-A) → $CD8^+$ *or* $CD4^+$ cells (CD8-PerCPCy5.5 v. CD4-AF700) → cytokine positive (SSC-A v. TNF-α-FITC *or* IFN-γ-APC *or* IL-2-PE). $Cytokine^+$ gates were set based on fully stained, PBS- or antigen-negative tumor cell line-stimulated controls.

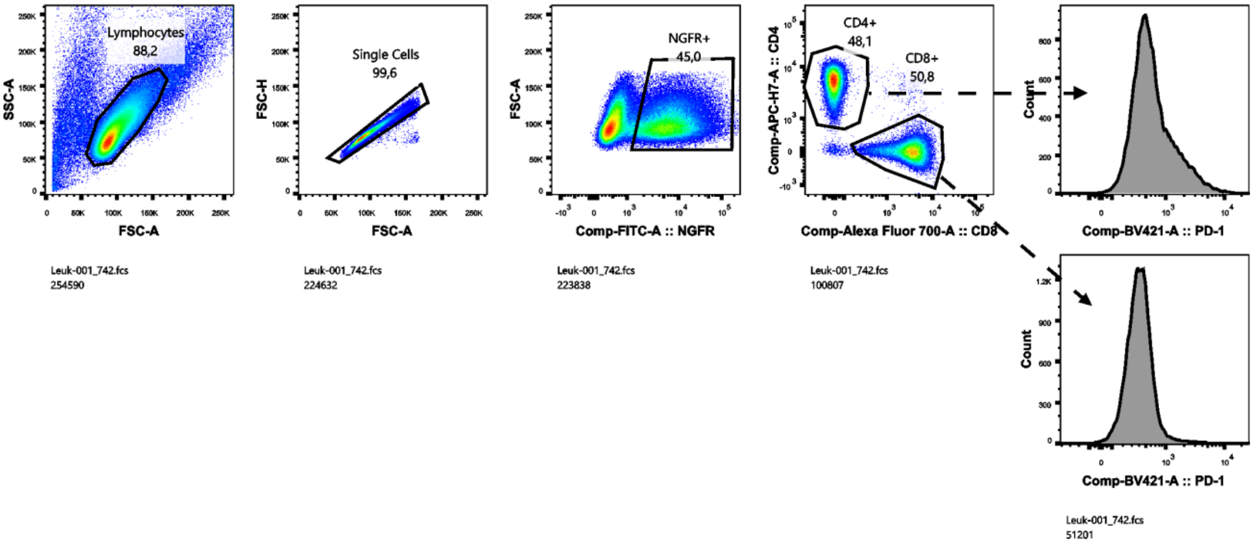

Supplementary Figure 14:

Examples of gating strategies used for the analysis of flow cytometry data. Gating strategy for checkpoint analysis of engineered T cells. Lymphocytes (SSC-A v. FSC-A) → singlets (FSC-H v. FSC-A) → NGFR$^+$ cells (SSC-A v. NGFR- VioBright FITC) → CD8$^+$ *or* CD4$^+$ cells (CD8-AF700 v. CD4-APC-H7-A) → Checkpoint receptor expression as a histogram (TIM-3-BV785/ LAG-3-AF647/ PD-1-BV421). NGFR+ gates were set based on fully stained, vector control transduced T cells.

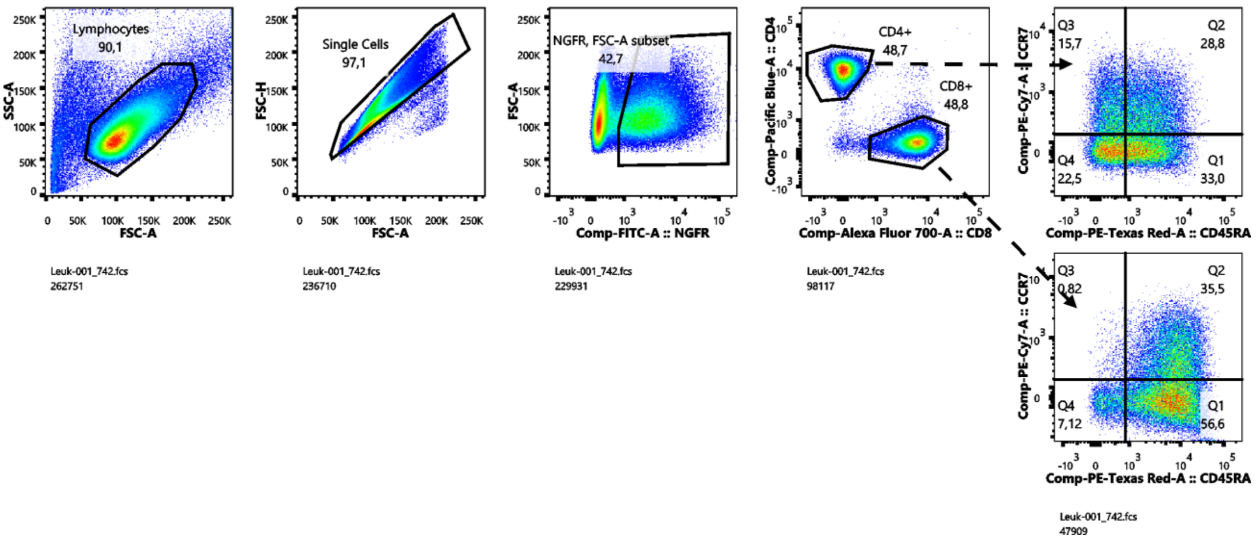

Supplementary Figure 15:

Examples of gating strategies used for the analysis of flow cytometry data. Gating strategy for memory population analysis of engineered T cells. Lymphocytes (SSC-A v. FSC-A) → singlets (FSC-H v. FSC-A) → NGFR$^+$ cells (SSC-A v. NGFR- VioBright FITC) → CD8$^+$ *or* CD4$^+$ cells (CD8-AF700 v. CD4- Pacific Blue) → Checkpoint receptor expression was analysed via quarter analysis (CD27-APC-H7/ CD28-PE/ CD45RA-ECD/ CCR7-PE-Cy7). NGFR$^+$ gates were set based on fully stained, vector control transduced T cells.

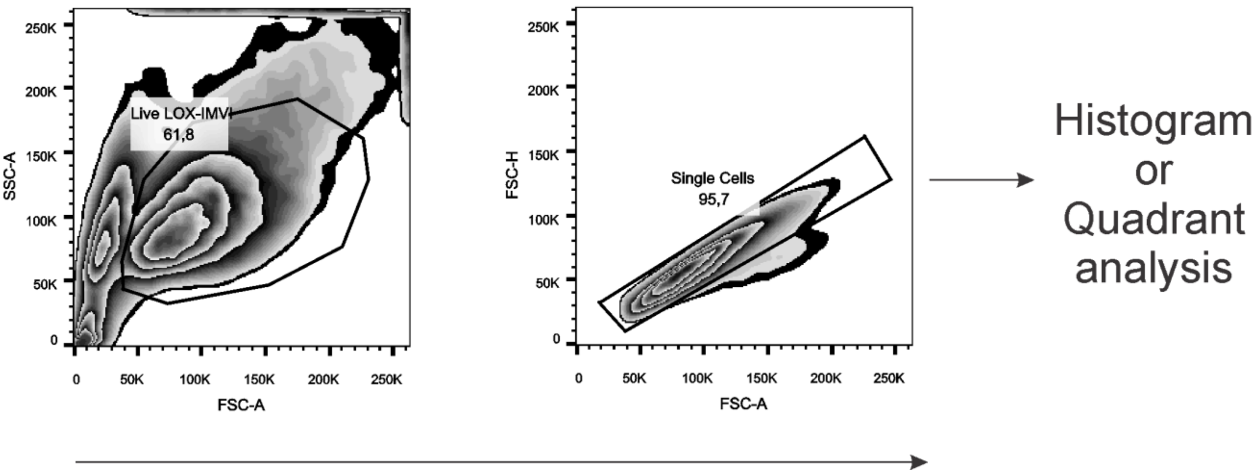

Supplementary Figure 16:

Examples of gating strategies used for the analysis of flow cytometry data. Gating strategy for various adherent cell lines engineered with various surface expressing proteins. Cell gate (SSC-A v. FSC-A) → singlets (FSC-H v. FSC-A) → Quadrant or Histogram analysis with relevant markers

Test for significance in cytokine levels

Day 3	Conditions	GM-CSF	IFNg	IL-1b	IL-2	IL-4	IL-6	IL-8	IL-10	IL-12 (p70)	MCP-1	TNFa	IL-13	IL-5
	TAC-Vector	NS	NS	NS	NS	NS	NS	NS	NS	NS	N/A	NS	NS	NS
	TAC-CAR	0.00068	0.00051	NS	< 0.0001	< 0.0001	NS	NS	0.00101	NS	N/A	0.0036	0.00239	NS
	CAR-Vector	0.00013	0.00021	NS	< 0.0001	< 0.0001	NS	NS	0.0009	NS	N/A	0.00254	0.00052	NS

Day 7	Conditions	GM-CSF	IFNg	IL-1b	IL-2	IL-4	IL-6	IL-8	IL-10	IL-12 (p70)	MCP-1	TNFa	IL-13	IL-5
	TAC-Vector	NS	NS	N/A	NS	NS	NS	NS	NS	NS	NS	0.00093	NS	NS
	TAC-CAR	< 0.0001	0.00035	N/A	0.00038	0.00522	NS	0.00296	0.00061	0.00652	NS	NS	0.00025	< 0.0001
	CAR-Vector	< 0.0001	0.00035	N/A	0.00035	0.00523	NS	0.00325	0.00063	0.00867	NS	NS	0.00025	< 0.0001

Supplementary Table 1:

Statistical analysis of serum cytokine data. An unpaired t-test was used to evaluate the statistical significance of the serum cytokine data (n = 3 for each of HER2-TAC-, HER2-CAR-, and vector control T cells) presented in Figure 6. Pairwise comparisons between the three treatment groups are presented for each of the thirteen cytokines tested. P-values are shown. NS = not significant using a confidence interval of 95%. N/A denotes cases where no analysis was mathematically possible.

FWD-1	GGCGCGCCATGGATTTCCAGGTCCAGATTTTC
REV-1	CCCGGGGTTCAGGTCTTCTTCGCTAATC
FWD-2	ATATGGCGCGCCATGGATTTCC
REV-2	GCTGAACTTCACTCTGCAGTAAAGGGTGATAACCAG
FWD-3	ATCACCCTTTACTGCAGAGTGAAGTTCAGCAGGAG
REV-3	ATATGCTAGCTTAGCGAGGGGG
FWD-4	ATATGGATCCGTGGGGTCACCGTCTCCTCAACC
REV-4	ATATGCTAGCTTAGCGAGGGGGCAGGGC
FWD-5	CATGTGACTCCACGGAGTACCGG
REV-5	GTCCGAACGTCCACGGCAGAG
FDW-6	GGTCTTCTTCGCTAATCAGTTTCTGTTCCGGCCTGGCGGCGTGG
REV-6	CCACGCCGCCAGGCCGGAACAGAAACTGATTAGCGAAGAAGACC

Supplementary Table 2:
Sequence of oligos used in construction of various plasmid constructs described in materials and methods.
